# Construction Notes

**Published:** 1894-07-14

**Authors:** 


### CONSTRUCTION NOTES.

### Rotunda Hospital Extensions.

A new auxiliary wing to the Dublin Rotunda Hospital is in course of construction. The plans are prepared by Mr. Albert E. Murray, R.H.A., of Dawson Street, and the contract was entrusted to Mr. James Kernan, of Talbot Street. The building is being erected on the western side of Rutland Square, the architectural style being the Georgian. The sanitary arrangements are having all due attention paid to them, and 60 beds, all devoted to the care of diseases peculiar to women, will be provided. The plans make ample provision for kitchen arrangements, and the operating theatre will be as complete in every particular as modern science could demand. There will be additional wards for private patients. The new wing will be of the same height as the Rotunda, the floors being on the same levels, and possessing direct connection with the hospital. The building is progressing, and will probably be completed in twelve months' time.

### Enlargement of the Female Workhouse Infirmary, Kingston.

The foundation-stone of the new wing of this infirmary has just been laid. The additional accommodation is estimated at a cost of £6,000, and will provide all that is necessary for many years to come. The new wing is to be built in the Gothic style, to correspond as nearly as possible with the existing buildings, and will contain six wards, three of them 27 feet by 24 feet, and three 39 feet by 24 feet. Three nurses' duty-rooms will be provided, bath-rooms, lavatories, &c., and thus accommodation will be made for seven additional nurses and sixty additional patients. The building will be faced with red bricks and Portland stone dressing and the roof will be slated. The first floor is to be approached by a flight of stone steps, and the upper floors by stone staircases on each end of the building. All the floors will be fireproof, and all angles of walls and ceilings circular, the intersections of walls with floors being formed with glazed concave bricks. The ventilation, heating, and sanitation are to be in accordance with the most approved modern principles. The arrangements of the walls will facilitate the proper classification of the sick. The architect who designed this much-needed extension to the already existing building was Mr. W. H. Hope, the contractors being Messrs. W. H. Lorden and Sons, of Upper Tooting.

### London Homœopathic Hospital.

The architect, Mr. W. A. Pite, F.R.I.B.A., writes to say, with reference to our comments on the plans which were published in The Hospital for June 30th, that the original plan of the ward bath-rooms has been altered, so as to meet the criticism we made thereon.

